# Diagnostic Performance of the Radiological Society of North America Consensus Statement for Reporting COVID-19 Chest CT Findings: A Revisit

**DOI:** 10.3390/jcm12165180

**Published:** 2023-08-09

**Authors:** Mohammed Hazem, Sayed Ibrahim Ali, Qasem M. AlAlwan, Ibrahim Khalid Al Jabr, Sarah Abdulrahman F. Alshehri, Mohammed Q. AlAlwan, Mohammed Ibrahim Alsaeed, Mohammed Aldawood, Jamela A. Turkistani, Yasser Abdelkarim Amin

**Affiliations:** 1 Department of Surgery, Collage of Medicine, King Faisal University, P.O. Box 400, Al-Ahsa 31982, Saudi Arabia; ialjabr@kfu.edu.sa (I.K.A.J.); saalshehri@kfu.edu.sa (S.A.F.A.); 2 Diagnostic and Interventional Radiology Department, Faculty of Medicine, Sohag University, Sohag 82524, Egypt; yasser_amin@med.sohag.edu.eg; 3 Department of Family and Community Medicine, Collage of Medicine, King Faisal University, P.O. Box 400, Al-Ahsa 31982, Saudi Arabia; seali@kfu.edu.sa (S.I.A.); jturkistani@kfu.edu.sa (J.A.T.); 4 Educational Psychology Department, College of Education, Helwan University, Cairo 11795, Egypt; 5 Department of Radiology, King Fahd Hospital Hofuf, Al-Ahsa 36441, Saudi Arabia; qalalwan@moh.gov.sa (Q.M.A.); mohammed.alalwan@hotmail.com (M.Q.A.); 6 King Abdulaziz Military Hospital, Al-Ahsa 36428, Saudi Arabia; mohammed.saeed20136@gmail.com; 7 Collage of Medicine, King Faisal University, P.O. Box 400, Al-Ahsa 31982, Saudi Arabia; mohammed.a.dawood19@gmail.com

**Keywords:** COVID-19, RSNA consensus, typical category, atypical category, indeterminate category, negative category

## Abstract

Coronavirus disease 2019 (COVID-19) is a highly contagious respiratory disease that leads to variable degrees of illness, and which may be fatal. We evaluated the diagnostic performance of each chest computed tomography (CT) reporting category recommended by the Expert Consensus of the Radiological Society of North America (RSNA) in comparison with that of reverse transcription polymerase chain reaction (RT-PCR). We aimed to add an analysis of this form of reporting in the Middle East, as few studies have been performed there. Between July 2021 and February 2022, 184 patients with a mean age of 55.56 ± 16.71 years and probable COVID-19 infections were included in this retrospective study. Approximately 64.67% (119 patients) were male, while 35.33% (65 patients) were female. Within 7 days, all patients underwent CT and RT-PCR examinations. According to a statement by the RSNA, the sensitivity, specificity, positive predictive value (PPV), negative predictive value (NPV), and accuracy of each CT reporting category were calculated, and the RT-PCR results were used as a standard reference. The RT-PCR results confirmed a final diagnosis of COVID-19 infection in 60.33% of the patients. For COVID-19 diagnoses, the typical category (*n* = 88) had a sensitivity, specificity, PPV, and accuracy of 74.8%, 93.2%, 94.3%, and 92.5%, respectively. For non-COVID-19 diagnoses, the PPVs for the atypical (*n* = 22) and negative (*n* = 46) categories were 81.8% and 89.1%, respectively. The PPV for the indeterminate (*n* = 28) category was 67.9%, with a low sensitivity of 17.1%. However, the RSNA’s four chest CT reporting categories provide a strong diagnostic foundation and are highly correlated with the RT-PCR results for the typical, atypical, and negative categories, but they are weaker for the indeterminate category.

## 1. Introduction

Coronavirus disease 2019 (COVID-19) is defined as an infectious viral disease caused by severe acute respiratory syndrome coronavirus 2 (SARS-CoV-2), which affects the respiratory system [1,2]. The disease was discovered in Wuhan, China in December 2019 and has rapidly spread throughout the world, leading to great scientific, economic, and social impacts [3]. Therefore, the World Health Organization declared the COVID-19 outbreak as an emergency for public health on 30 January 2020 [4]. It was declared that the situation was no longer that of a pandemic in May 2023 in an announcement by a WHO chief; as a result, COVID-19 was no longer considered a global health emergency [5].

The clinical symptoms of COVID-19 vary, and can include no symptoms or mild symptoms (generalized body ache, nasal congestion, fever, dry cough, and altered sense of taste or smell) [6,7]. In severe cases, the infection can cause dyspnea, pneumonia, hypoxemia, severe acute respiratory syndrome, coagulopathy, and metabolic abnormalities, and may result in death [4,8,9].

The gold standard technique for diagnosis is reverse transcription polymerase chain reaction (RT-PCR), which is performed on respiratory secretions [10,11,12]. However, RT-PCR has been reported to have only moderate sensitivity, ranging from 60% to 71%, with many false negative cases [13]. These false negative results could be due to a low initial viral load; therefore, repeating the test is necessary in many cases [13,14]. In addition, the results take a long time to materialize—from 6 h to 48 h—which is not suitable for emergency situations [15].

Radiological examination is considered a very important complementary investigation in diagnosis and follow-up, especially in RT-PCR-negative cases. The first radiological examination is chest radiography; however, in the early stages of the disease, the observations appear normal. Chest CT is the best radiological tool for examining a suspected COVID-19 infection due to its sensitivity in detecting infection-induced pneumonic changes [15]. In addition, chest CT can be used to detect peripheral areas of ground-glass opacity (GGO), which is a characteristic sign of early COVID-19 [15].

Moreover, a CT scan is useful in assessing disease progression or evaluating the response to treatment [16], and provides immediate or rapid results [17]. Chest CT can be used to display a group of characteristic findings, including areas of GGO that are or are not associated with consolidation, signs of reticulation (crazy paving pattern), vascular changes, and reversed halo signs [14,18,19,20].

Multiple studies have confirmed that chest CT findings appear earlier than symptoms, which makes the role of imaging very important for early diagnosis, rapid isolation, and prevention of the disease’s spread [21,22,23,24]. To help and guide radiologists in creating uniform reports, the Radiological Society of North America (RSNA) has proposed a language of chest CT reporting that includes four categories: typical, indeterminate, atypical, and negative [25].

This study aimed to assess the diagnostic performance of each category of the Expert Consensus of the RSNA for chest CT reporting for rapid treatment in comparison with RT-PCR in the Middle East.

## 2. Methodology

### 2.1. Study Design

We enrolled 184 patients in this observational single-center retrospective study between July 2021 and February 2022. Approval was obtained from our Institutional Review Board (IRB) and the relevant ethics committee (H-05-HS-065), and the study was conducted in conformity with the Helsinki Declaration and its later amendments. The requirement for informed written consent was waived due to the retrospective nature of this study. All of the information was kept confidential.

#### 2.1.1. Inclusion Criteria

1. Clinically suspected COVID-19 infection, including
  - Fever with one or more of the following symptoms: dyspnea, cough, and sore throat.
  - Contact with a positive case of COVID-19 infection and exhibition of mild symptoms.
2. Chest CT scan performed after admission.
3. Duration of the interval between the CT examination and RT-PCR of less than 7 days.

#### 2.1.2. Exclusion Criteria

(a) No RT-PCR test results for COVID-19.
(b) The interval between RT-PCR and the CT examination was more than 7 days.
(c) Poor chest CT images (presence of a motion artifact).
(d) Presence of chronic respiratory disease (for example, pulmonary tuberculosis, interstitial lung fibrosis, or chronic obstructive pulmonary disease).

**Figure 1:**
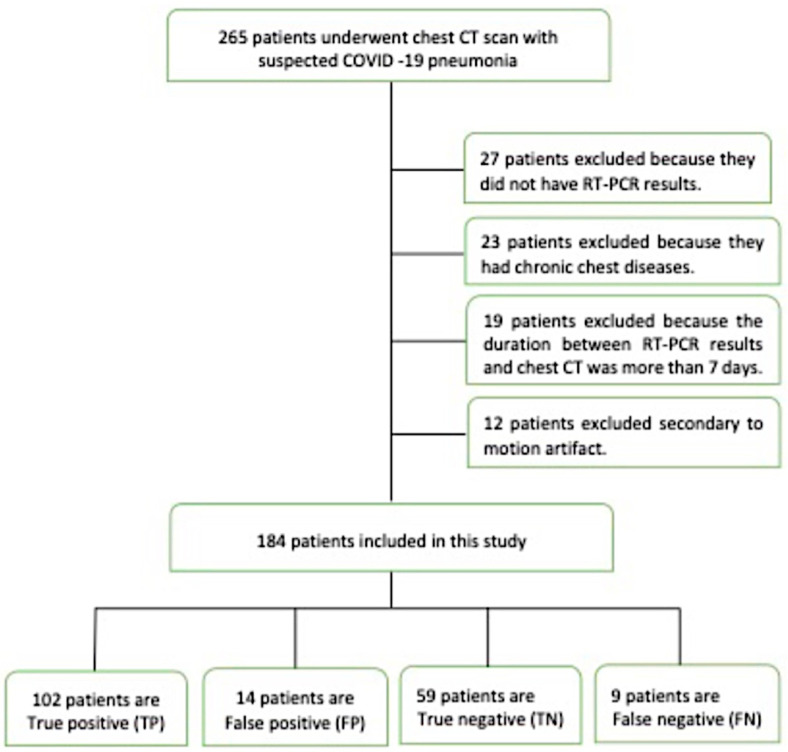
Flowchart of the study; RT-PCR means reverse transcription polymerase chain reaction.

Figure 1 depicts the flowchart of this study.

### 2.2. Clinical Data

The electronic medical records of all included patients were reviewed. Moreover, clinical information, including sex, age, patient symptoms and signs, dates and results of the RT-PCR tests, dates of the chest CT examinations, and any comorbidities, including hypertension, diabetes, and chronic abdominal diseases (referring to any organic abdominal disease that extended more than 3 months, including diseases such as chronic liver, kidney, gastric, and bowel diseases as well as abdominal malignancies) were recorded.

### 2.3. CT Acquisition Technique

All chest CT examinations were conducted in the supine position without contrast and for one full inspiratory breath. In the study, two CT machines were used. The first was a GE Discovery CT750 HD 128 slice (General Electric Healthcare, Boston, MA, USA) with the following typical technical scan parameters: tube current modulation, 219 mAs; tube voltage, 140 kV; spiral pitch factor, 0.9–1.37; matrix, 512 × 512; collimation width, 64 × 0.625. All of the images were reconstructed with a slice thickness and interval of 1 mm.

The second CT machine was a Siemens Somatom Definition AS+ 128 slice (Siemens Healthcare, Erlangen, Germany). The average technical scan parameters were as follows: tube current modulation (mA) with automatic exposure control; tube voltage, 120 kV; spiral pitch factor, 1.2; matrix, 512 × 512; collimation width, 64 × 0.6. All of the images were reconstructed with a slice thickness and interval of 1 mm.

After examining each patient, strict disinfection precautions were followed according to the department’s guidelines.

### 2.4. Image Analysis

Two senior radiologists each with more than 10 years of experience in interpreting chest CT images were blinded to each other as well as to the RT-PCR results. The CT findings were independently evaluated initially in axial and then in multiplanar reconstruction images. Decisions were reached by consensus.

**Figure 2:**
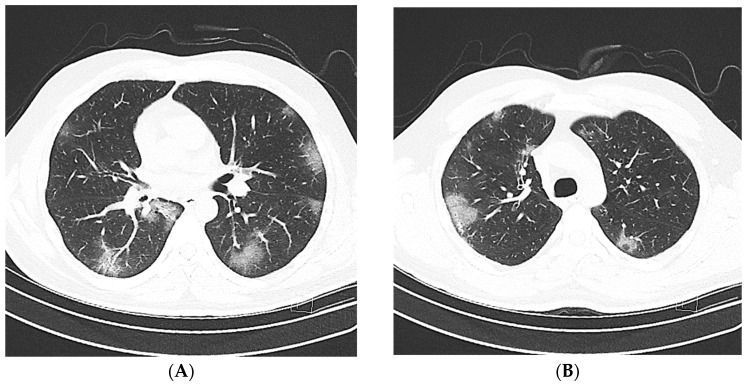

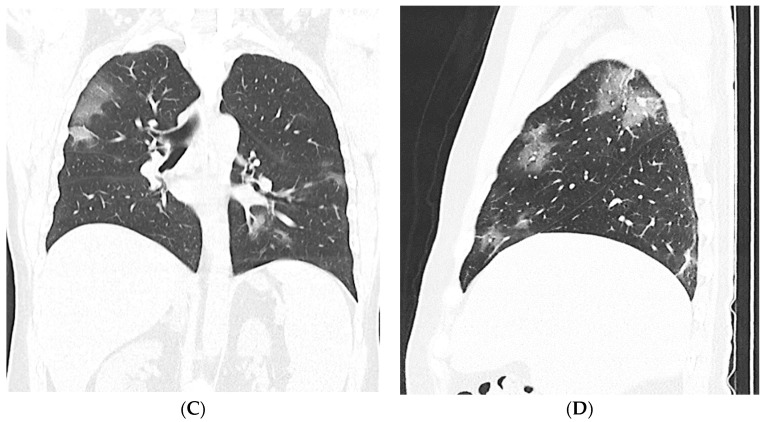
Typical chest CT imaging findings for COVID-19. Non-contrast thin-section axial images (**A**,**B**), a coronal multiplanar reformatted image (**C**), and a sagittal multiplanar reformatted image (**D**) of the lungs of a 51-year-old male with positive RT-PCR, displaying multiple bilateral ground-glass opacities with a peripheral predominance, many with a round morphology.

**Figure 3:**
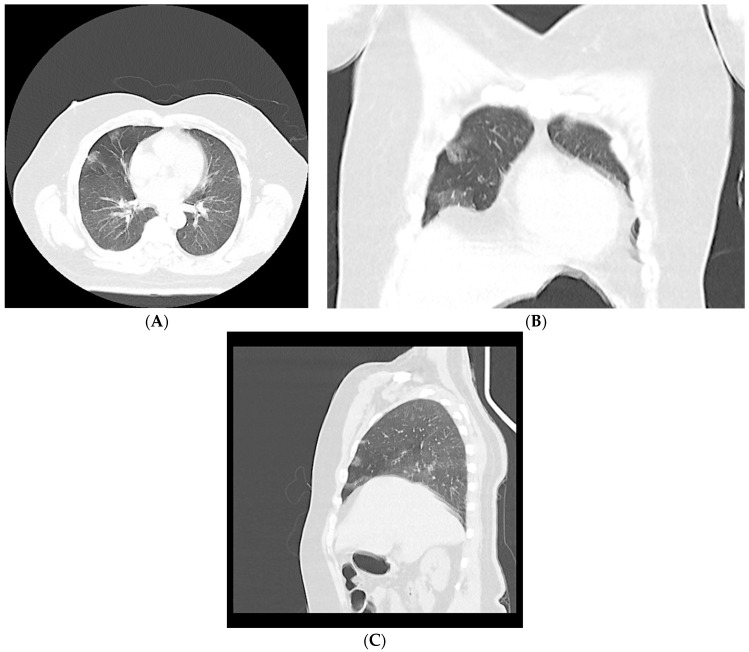
Typical chest CT imaging findings for COVID-19. A non-contrast thin-section axial image (**A**), a coronal multiplanar reformatted image (**B**), and a sagittal multiplanar reformatted image (**C**) in a 59-year-old male with positive RT-PCR displaying multiple bilateral, peripheral, and nearly rounded ground-glass opacities.

**Figure 4:**
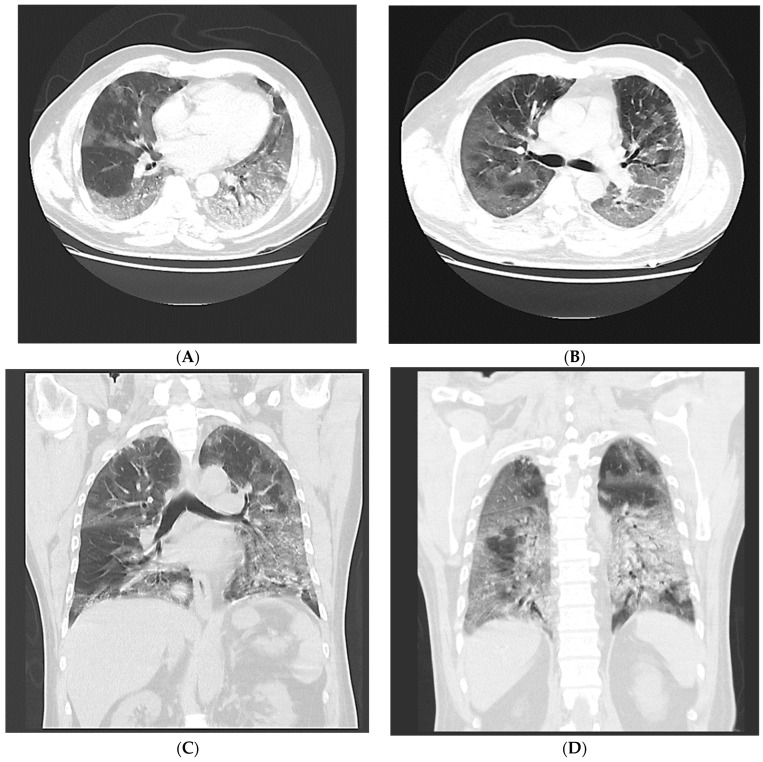
A 63-year-old male with positive RT-PCR. Non-contrast thin-section axial (**A**,**B**) and coronal multiplanar reformatted (**C**,**D**) images of the lung exhibiting diffuse ground-glass opacity without a specific distribution, i.e., an “indeterminate” pattern.

**Figure 5:**
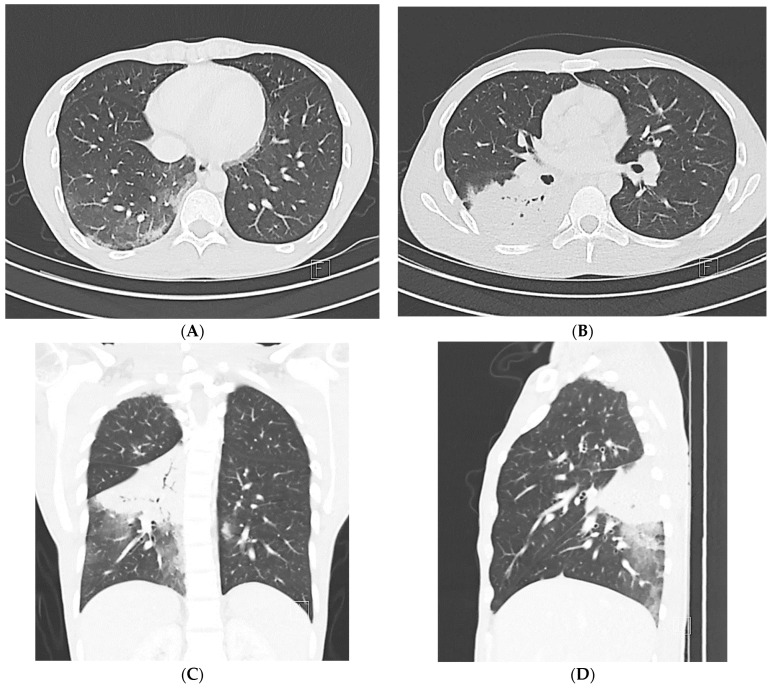
Indeterminate CT imaging features of COVID-19. Non-contrast thin-section axial images (**A**,**B**), a coronal multiplanar reformatted image (**C**), and a sagittal multiplanar reformatted image (**D**) of the lungs of a 57-year-old male with positive RT-PCR, exhibiting right-sided lower lobe superior segment consolidation with multiple adjacent areas of ground glass opacities.

**Figure 6:**
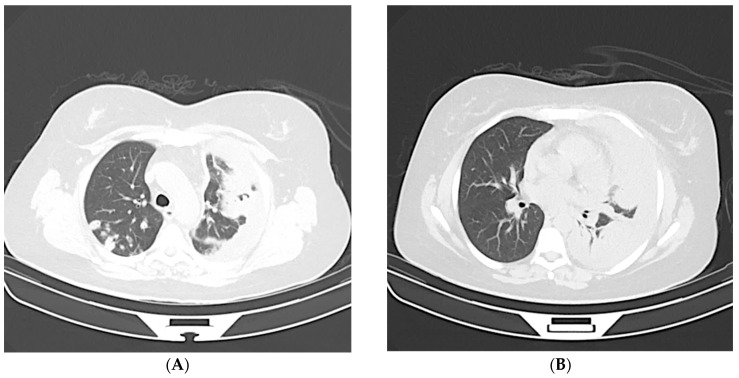

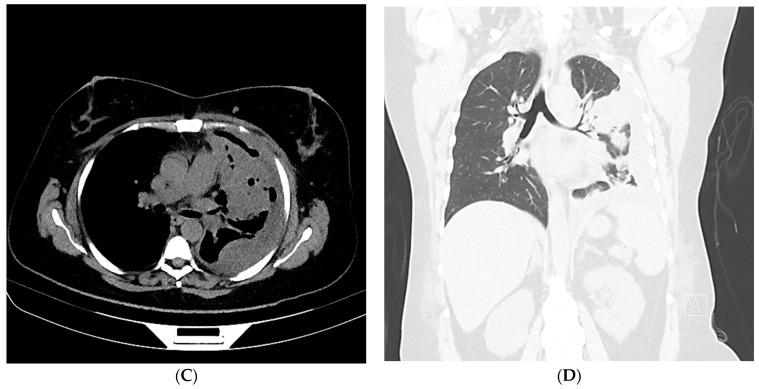
Atypical chest CT imaging findings for COVID-19. A non-contrast thin section axial image lung window (**A**,**B**), axial mediastinal window (**C**), and coronal multiplanar reformatted image (**D**) of the lungs of a 56-year-old female with negative RT-PCR. The images depict a large area of consolidation, with an air-bronchogram in the left lung, left pleural effusion, multiple small nodules with a tree-in-bud appearance in the right upper lobe, and small hilar lymph nodes, a pattern suggestive of occupational pneumonia.

The patients were classified into CT-negative and CT-positive groups based on the existence of CT findings that were highly suggestive of a COVID-19 infection according to the consensus statement of the Society of Thoracic Radiology (STR)/American College of Radiology (ACR)/RSNA [25]. Positive CT scans were classified as typical or indeterminate, whereas negative CT scans were classified into the atypical or negative categories (Figure 2, Figure 3, Figure 4, Figure 5 and Figure 6).

The detailed evaluation and analysis of the chest CT images were based on the following criteria: (a) unilateral or bilateral involvement; (b) distribution of the lesions: peripheral (lesions within the outer third), central (lesions in the inner two-thirds) [26], or mixed distribution; (c) pattern of the lesion: pure GGO, consolidation, mixed GGO, reversed halo sign, interlobular tissue thickening, bronchial dilatation, tree-in-bud sign, pleural effusion, peribronchial thickening, crazy-paving pattern, vascular enlargement, lymphadenopathy, and any other radiological signs.

### 2.5. RT-PCR Results

RT-PCR data were collected from the patients’ files that were saved in our hospital information system. Most patients underwent an RT-PCR test only once (147 patients); however, the remaining patients underwent the test multiple times (37 patients) because the initial results were negative. A COVID-19 infection was confirmed when the RT-PCR test result was positive. The RT-PCR results were utilized as the benchmark, on which basis we divided our patients into the following:

- True positive (TP): patients with positive RT-PCR results and typical or indeterminate CT categories.
- False positive (FP): patients with negative RT-PCR results and typical or indeterminate CT categories.
- True negative (TN): patients with negative RT-PCR results and atypical or negative CT categories.
- False negative (FN): patients with positive RT-PCR results and atypical or negative CT categories.

### 2.6. Statistical Analysis

All of the data were collected and analyzed using the SPSS^®^ program (version 26, Statistical Package for Social Science). Depending on the type of data, information is presented as either the mean and standard deviation or the frequency and percentage. The Chi-square test was used to determine the relationship between the two categorical variables. The results were considered significant at *p* < 0.05.

Using the RT-PCR data as a standard of reference, the sensitivity, specificity, PPV, NPV, and accuracy of each chest CT category, age group, and sex group were measured, along with their 95% confidence intervals (CIs).

We utilized the Cohen’s kappa test to determine the interobserver variability, using the following scale:

- Slight agreement: between 1% and 20%
- Fair agreement: between 21% and 40%
- Moderate agreement: between 41% and 60%
- Significant agreement: between 61% and 80%
- Nearly perfect or perfect agreement: between 81% and 100%.

## 3. Results

### 3.1. Patients’ Demographic and Clinical Data

A total of 265 patients were admitted to our emergency department with a clinical suspicion of COVID-19. Of these, 27 patients were excluded because they had no RT-PCR results, 23 patients were excluded because of chronic respiratory disease, 19 were excluded because the interval between the chest CT and RT-PCR results was more than 7 days, and 12 patients were excluded due to motion artifacts in their chest CT scans (Figure 1).

After exclusion, 184 patients were included, with a mean age of 55.56 ± 16.71 years (range: 18–95 years). Among the total patients, approximately 64.67% (119 patients) were males, while females represented approximately 35.33% (65 patients).

On the other hand, among all of the patients, 73 patients (39.67%)—with a mean age of 52.22 ± 16.44 years—had negative RT-PCR test results, while 111 patients (60.33%)—with a mean age of 57.77 ± 16.58 years—had positive results. The median time gap between the RT-PCR analysis and chest CT scan was 3 days (range: 1–7 days). Table 1 lists all of the demographic and clinical characteristics.

Regarding patients with chronic abdominal diseases, there were no statistically significant differences between patients with positive PCR results and those with negative results.

### 3.2. Analysis of the CT Findings

Patients with probable COVID-19 infections exhibited various chest CT abnormalities. To describe the chest CT findings for each group, we divided these patients into two categories based on their RT-PCR results, i.e., positive and negative.

The following were more prevalent and differed significantly in patients with positive RT-PCR results: pure GGO, consolidation, mixed GGO, bilateral distribution, vascular enlargement, reversed halo sign, and crazy paving pattern (*p* < 0.0001).

In contrast, pleural effusion, central distribution, and bronchial dilatation were more common in patients with negative RT-PCR results, with no significant differences (*p* > 0.05). Moreover, tree-bud appearance was significantly more common in patients with negative RT-PCR results (*p* < 0.0001). All detected chest CT findings are summarized in Table 2.

### 3.3. Correlation of the CT Categories with the PCR Results

The chest CT findings were classified as typical, indeterminate, atypical, or negative using the consensus statement of the STR/ACR/RSNA, and these categories were correlated with the RT-PCR results.

Regarding the group that was determined to be positive according to the consensus statement of the STR/ACR/RSNA (including the typical and indeterminate categories), 83 of 88 patients with a typical appearance had positive RT-PCR results, while the remaining five patients had negative RT-PCR results, and there was a significant difference (*p* < 0.0001). The indeterminate category demonstrated non-significant differences between patients with RT-PCR-positive results and those with negative results (*p* = 0.071).

For the negative group, which included the atypical and negative categories, 18 of 22 patients with an atypical appearance had negative RT-PCR results, while the remaining four patients had positive RT-PCR results, and there was a statistically significant difference (*p* < 0.018). In addition, the negative group included 46 patients; only 5 had positive RT-PCR results, while the remaining 41 had negative RT-PCR results, with a statistically significant difference (*p* < 0.001). Table 3 displays all of the data regarding the chest CT categories and RT-PCR results.

### 3.4. Diagnostic Performance of the CT Categories

Using the results of RT-PCR as a standard reference and comparing them with the findings on the chest CT categories, 102 patients were identified to be TPs, 59 were TNs, 14 were considered FPs, and 9 were FNs.

The statistical analysis of the diagnostic performance of all groups demonstrated that the typical category (*n* = 88) had a sensitivity of 74.8%, a specificity of 93.2%, a PPV of 94.3%, and an accuracy of 92.5% for COVID-19. The indeterminate pattern (*n* = 28) demonstrated low sensitivity (17.1%), good specificity (87.7%), a PPV of 67.9%, and a low accuracy (45.1%) for COVID-19. Combining the typical and indeterminate groups to measure the diagnostic performance of the chest CT examinations in detecting positive cases revealed a high sensitivity of 91.9%, a specificity of 80.8%, a PPV of 87.9%, an NPV of 86.8%, and an accuracy of 90.7%.

Additionally, the atypical category displayed a high specificity of 96.4%, a PPV of 81.8%, and an accuracy of 82.07%, while the negative category also demonstrated a high specificity of 95.5%, a PPV of 89.1%, and an accuracy of 79.9%.

Data regarding the diagnostic performance of all chest CT categories are listed in Table 4.

The agreement between the two radiologists was perfect for the typical and negative patterns (95.2% and 98.7%, respectively), with significant agreement for the indeterminate (78.8%) and atypical (76.6%) patterns.

### 3.5. The Diagnostic Performance of Chest CT Examination for Each Age Group and Sex Compared to the RT-PCR Results as a Gold Standard

Using the outcomes of RT-PCR as a reference, we conducted additional analyses to evaluate the diagnostic performance of chest CT examinations across sexes and age groups (Table 5).

In terms of diagnostic performance (sensitivity, specificity, PPV, NPV, and accuracy), we could not identify any statistically significant differences (*p* = 0.056, 0.535, 0.08, 0.298, and 0.136, respectively) between the age groups (<50 years and ≥50 years).

Similarly, there were no statistically significant differences in specificity, PPV, or accuracy between males and females (*p* = 0.86, 0.428, and 0.184, respectively); however, there were statistically significant differences in sensitivity and NPV (*p* = 0.002 and 0.001, respectively).

The RT-PCR-positive rate was 52.86% in patients aged < 50, 64.91% in patients aged ≥ 50, 55.46% in males, and 69.23% in females.

## 4. Exploring FPs, FNs, and TNs

Based on our research, we discovered that fourteen patients had FP results. All of them were admitted, separated into special rooms, and treated as patients with COVID-19 according to clinical suspicion, laboratory findings, and chest CT findings until they were discharged (twelve patients) or passed away (two patients). The patients were admitted to the hospital for approximately 10–17 days.

Further, nine patients had FN results, and seven of them displayed mild clinical manifestations, whereas the remaining two exhibited moderate clinical manifestations of COVID-19. All patients were treated and discharged after improvement; they were admitted to the hospital for approximately 25–34 days.

Fifty-nine patients had TN results; two of them had a final diagnosis of pulmonary embolism, four patients were diagnosed with chronic heart failure, and the remaining fifty-three patients were treated for other causes of pneumonia, with clinical improvement and recovery in forty-five patients and death in three patients. The clinical data of the remaining eleven patients were unavailable, as they completed their treatment outside the hospital (referred to as home treatment).

## 5. Discussion

Multiple findings have been detected in chest CT examinations of patients with COVID-19 infections since the first appearance of the disease in China. While most chest CT findings have been repeated elsewhere, an accurate diagnosis ultimately depends on the reader’s experience [27].

Additionally, a portion of individuals may exhibit persistent reticulations or diffuse alveolar destruction, while others may display bronchial deformities in the chronic stage of the disease, making it difficult to interpret their chest CT data [28,29].

Therefore, providing a standardized approach for reporting COVID-19 cases is very important. The RSNA, with contributions from the STR and ACR, formed an expert team to solve this matter and provided the best method in order to guide radiologists in coherently reporting their CT findings. However, reproducibility and reliability are necessary in order for this process to become widespread [27,30].

In our study, we analyzed the chest CT findings of 184 patients who met our inclusion criteria and had a clinically suspected COVID-19 infection. According to the agreement of the expert panel of the RSNA, the participants were split into four groups. The typical group demonstrated high diagnostic accuracy, with a specificity of 93.2% and an accuracy of 92.5%. Similarly, the negative category displayed high diagnostic performance, with a specificity of 95.5% and accuracy of 79.7%, and the atypical group exhibited a specificity of 96.4% and an accuracy of 82.07%. In contrast, the indeterminate category displayed worse diagnostic performance, with a sensitivity of 17.1%, a specificity of 87.7%, and an accuracy of 45.1%.

Furthermore, we discovered that the typical, atypical, and negative categories were strongly related to the results of RT-PCR, with the typical pattern having the highest PPV (94.3%) for COVID-19 infection, while the atypical and negative patterns had PPVs of 81.1% and 89.1%, respectively, for non-COVID-19 infections. Determining atypical and negative patterns is important because they can be produced by other causes.

Our results agree with those obtained by Ciccarese et al. for the typical, atypical, and negative categories; they discovered that the typical group had a PPV of 87.8%, sensitivity of 71.6%, and specificity of 91.6%, whereas the atypical and negative groups had PPVs of 89.6% and 86.2%, respectively. In contrast, our results regarding the indeterminate group were comparatively higher, as we detected a higher PPV of 67.9% than that reported in the results of Ciccarese et al. (36.7%) [31].

In concordance with our results, Som et al. reported an accuracy of 85.4% for the typical group, which was similar to our results, and a PPV of 81.1%, which was less than that in our results. On the other hand, the indeterminate group demonstrated an accuracy of 46%, which was similar to our result of 45.1%, while our PPV of 67.9% was greater than the PPV of 25% reported by Som et al. [30].

Moreover, our findings concur with those of studies that reported high sensitivity of CT for COVID-19 pneumonia, as we observed that the positive categories (typical and indeterminate) had an average sensitivity of 91.9%, a specificity of 80.8%, a PPV of 87.9%, and an accuracy of 90.7%, which matched previously reported findings [16,32]. However, the atypical findings alone had a specificity of 96.4%, and the negative category had a high specificity of 95.5%; meanwhile, the PPVs for both were 81.8% and 89.1%, respectively. This implies that the negative categories did not exclude COVID-19 infection. These results are in line with the conclusions of previous studies, which have claimed that the negative group could not exclude COVID-19 infection because chest CT scans might appear normal in early stages or asymptomatic cases [30,33].

Rocha et al. discovered that the typical category, as estimated for RT-PCR-positive results, demonstrated a specificity of 98.5%, which was more than in our results; however, the PPV and NPV were very close to those in our results, as they observed a PPV of 97.3% and an NPV of 67.9%. In addition, the indeterminate category demonstrated a sensitivity of 36.1% and NPV of 56.2%, which were greater than we established, while their result regarding PPV was 67.9%, which is similar to our results. Moreover, they discovered that chest CT examination had high diagnostic accuracy in identifying individuals with COVID-19; they reported a sensitivity of 88.3%, a specificity of 79%, a PPV of 80.5%, and an NPV of 87.4%, in line with our results. Furthermore, the negative group (atypical and negative categories) displayed a specificity, PPV, and NPV that were similar to our findings; however, the sensitivity was higher than that in our results for the atypical group and lower than ours for the negative group [34].

Furthermore, the analysis of the interobserver agreement revealed that while typical and negative patterns were simple to recognize, the interpretation of indeterminate and atypical patterns required more skill. As a result, pattern identification should be handled by highly experienced radiologists.

Our study demonstrated high agreement between the two readers for the typical and negative categories (95.2% and 98.7%, respectively), whereas there was substantial agreement for the indeterminate and atypical categories (78.8% and 76.6%, respectively). This is in accordance with the results obtained by Silva et al. [27].

Finally, we conclude that chest CT could play a crucial complementary role with respect to RT-PCR for rapid patient management, as CT results are readily available with a high accuracy of 90.7%; this result is in agreement with the conclusions of Dangis et al. [35].

The difference between our results and those of others could be explained by the differences in the sample size, the number of negative RT-PCR results included in each study, the inclusion or exclusion of patients with chronic chest disease, the sampling technique, and the protocol used in the chest CT examinations.

In this study, we evaluated the diagnostic accuracy of chest CT for various age groups and sex, observing no statistically significant differences between age groups; however, significant differences in sensitivity and specificity were observed between males and females. This is in agreement with the results obtained by Metha et al., who identified no statistically significant differences for the age groups of <60 and ≥60 years [36]. However, it is in disagreement with the findings of Falaschi et al. [37], who observed a significant difference in sensitivity and specificity for patients who were <50 and ≥50 years of age. Moreover, our results disagree with those of Metha et al. and Falaschi et al. [36,37] regarding the difference in diagnostic performance between males and females, which could be explained by the difference in the number of patients in different age groups and the inclusion of patients with chronic chest disease.

Our study had several limitations. First, this was a single-center study, although we included a large number of patients. Second, the study was a retrospective analysis, meaning that in most cases we were unable to determine the duration of the interval between chest CT examination and the onset of clinical signs. Third, RT-PCR was employed as the reference standard; however, it has low sensitivity and a false negative rate of 30% to 40% [32,38], possibly due to low viral loads, processing and technical errors, manufacturer variations, and improper sampling [39]; the sensitivity of RT-PCR is highest from the fifth to the seventh day, and decreases before the fifth and after the seventh day of infection [40,41]. Fourth, many of the patients involved in this study were severely ill with typical symptoms and signs of COVID-19 infection. Fifth, there are a limited number of studies evaluating the diagnostic accuracy of the expert consensus of the RSNA available for comparison with our results.

A strength of our study is its comparatively large number of patients with negative RT-PCR results for COVID-19, which allowed for a more thorough examination of the RSNA’s guidelines.

## 6. Conclusions

The utilization of the four diagnostic categories suggested by the RSNA demonstrated greater diagnostic performance for most patients with COVID-19. These diagnostic categories can be a crucial tool in clinical decision-making and rapid treatment to complement RT-PCR testing.

## Figures and Tables

**Table 1:** Demographic and clinical features of patients with suspected COVID-19 infection (184 patients).

Variable	Total	RT-PCR + VE	RT-PCR − VE	*p*-Value
Demographic data				
Age (mean ± STD)	55.56 ± 16.7	57.77 ± 16.58	52.22 ± 16.44	0.027
Male	119 (64.67%)	66 (55.46%)	53 (44.54%)	0.329
Female	65 (35.33%)	45 (69.23%)	20 (30.77%)	0.012
Clinical data				
Hypertension	79 (42.93%)	48 (60.76%)	31 (39.24%)	0.247
DM	82 (44.56%)	64 (78.05%)	18 (21.95%)	0.001
Fever	111 (60.33%)	77 (69.37%)	34 (30.63%)	0.001
Chronic abdominal diseases	49 (26.63%)	29 (59.18%)	20 (40.82%)	0.402
Dry cough	115 (62.5%)	62 (53.91%)	53 (46.09%)	0.413
Expectoration	52 (28.26%)	28 (53.85%)	24 (46.15%)	0.512
Chest pain	32 (17.39%)	18 (56.25%)	14 (43.75%)	0.216
Fatigue	34 (18.48%)	18 (52.94%)	16 (47.06%)	0.371
Sore throat	57 (30.98%)	34 (59.65%)	23 (40.35%)	0.246
Insomnia	23 (12.5%)	12 (52.17%)	11 (47.83%)	0.811
Loss of taste	86 (46.74%)	64 (74.42%)	22 (25.58%)	0.0001
Dyspnea	42 (22.83%)	31 (73.81%)	11 (26.19%)	0.0132
Nausea/vomiting	42 (22.83%)	20 (47.62%)	22 (52.38%)	0.831
Diarrhea	29 (15.76%)	15 (51.72%)	14 (48.28%)	0.887

RT-PCR, reverse transcription polymerase chain reaction; STD, standard deviation; *p*-value significant at <0.05; COVID-19, coronavirus disease 2019.

**Table 2:** Chest CT features in patients with suspected COVID-19 infection (184 patients).

Variable		Total	RT-PCR + VE	RT-PCR − VE	*p*-Value
Pure GGO		34 (18.48%)	31 (91.18%)	3 (8.82%)	0.0001
Mixed GGO		82 (44.57%)	71 (86.59%)	11 (13.41%)	0.0001
Consolidation		104 (56.52%)	75 (72.12%)	29 (37.88%)	0.0001
Distribution	Unilateral	17 (9.24%)	9 (52.94%)	8 (47.06%)	0.637
	Bilateral	121 (65.76%)	107 (88.43%)	14 (11.57%)	0.0001
Vascular enlargement		56 (30.43%)	51 (91.07%)	5 (8.93%)	0.0001
Pleural effusion		14 (7.6%)	6 (42.86%)	8 (57.14%)	0.581
Interlobular septal thickening		64 (34.78%)	48 (75%)	16 (25%)	0.0001
Localization	Central	14 (7.6%)	6 (42.86%)	8 (57.14%)	0.581
	Peripheral	39 (21.19%)	35 (89.74%)	4 (10.26%)	0.0001
	Mixed	85 (46.2%)	64 (75.29%)	21 (24.71%)	0.0001
Peri-bronchial thickening		30 (16.3%)	17 (56.67%)	13 (43.33%)	0.553
Tree-in-bud		33 (17.93%)	4 (12.12%)	29 (87.88%)	0.0001
Reversed halo sign		25 (13.59%)	22 (88%)	3 (12%)	0.0001
Bronchial dilatation		11 (5.98%)	3 (27.27%)	8 (72.73%)	0.137
Crazy-Paving pattern		49 (26.63%)	43 (87.76%)	6 (12.24%)	0.0001

RT-PCR, reverse transcription polymerase chain reaction; *p*-value significant at <0.05; COVID-19, coronavirus disease 2019; GGO, ground glass opacity.

**Table 3:** CT category findings according to the RSNA/ACR/STR classification compared with RT- PCR results (184 patients).

CT Category	Number	RT-PCR Positive	RT-PCR Negative	*p*-Value
Typical appearance	88	83	5	0.0001
Indeterminate appearance	28	19	9	0.071
Atypical appearance	22	4	18	0.018
Negative	46	5	41	0.0001

RSNA/ACR/STR, Radiological Society of North America/Society of Thoracic Radiology/American College of Radiology; RT-PCR, reverse transcription polymerase chain reaction; *p*-value significant at <0.05.

**Table 4:** Diagnostic performance of chest CT categories according to RSNA/ACR/STR classification compared with the RT-PCR results as a standard reference (184 patients).

CT Categories	Number	Sensitivity % (95% CI)	Specificity % (95% CI)	PPV % (95% CI)	NPP % (95% CI)	Accuracy % (95% CI)
Typical appearance	88	74.8 (65.7–82.5)	93.2 (84.7–97.7)	94.3 (87.6–97.5)	70.8 (63.7–77.1)	92.5 (88.8–95.3)
Indeterminate appearance	28	17.1 (10.6–25.4)	87.7 (77.9–94.2)	67.9 (50.3–81.5)	41 (38.1–44)	45.1 (37.8–52.6 6)
Both (typical and indeterminate)	116	91.9 (85.2–96.2)	80.8 (69.9–89.1)	87.9 (81.9–92.1)	86.8 (77.6–92.5)	90.7 (87.7–93.2)
Atypical appearance	22	24.7 (15.3–36.1)	96.4 (91.0–99.0)	81.8 (61.3–92.7)	66.1 (60.7–74.6)	82.07 (75.7–87.3)
Negative	46	56.2 (44.1–67.8)	95.5 (89.8–98.5)	89.1 (77.3–95.2)	76.8 (71.8–81.2)	79.9 (73.4–85.4)

RSNA/ACR/STR, Radiological Society of North America/Society of Thoracic Radiology/American College of Radiology; RT-PCR, reverse transcription polymerase chain reaction; CI, confidence interval; PPV, positive predictive value; NPV, negative predictive value.

**Table 5:** Diagnostic performance of chest CT for each age group and sex compared with RT-PCR (184 patients).

Groups		Number	Sensitivity % (95% CI)	*p*-Value	Specificity % (95% CI)	*p*-Value	PPV % (95% CI)	*p*-Value	NPP % (95% CI)	*p*-Value	Accuracy % (95% CI)	*p*-Value
Age	<50	70	86.5 (71.2–95.5)	0.056	78.8 (61.1–91.0)	0.535	82.1 (70.1–89.9)	0.08	83.9 (69.3–92.3)	0.298	82.9 (72.0–90.8)	0.136
	≥50	114	94.6 (86.7–98.5)		82.5 (67.5–98.5)		90.9 (83.6–95.2)		89.2 (75.9–95.6)		90.4 (83.4–95.1)	
Sex	Males	119	97 (89.5–99.6)	0.002	81.1 (68.0–90.6)	0.86	86.5 (78.5–91.8)	0.428	95.6 (84.5–98.8)	<0.0001	89.9 (83.1–94.7)	0.184
	Females	65	84.4 (70.5–93.5)		80 (56.3–94.3)		90.5 (79.7–95.8)		69.6 (52.8–82.4)		83.1 (71.7–91.2)	

RT-PCR, reverse transcription polymerase chain reaction; CI, confidence interval; PPV, positive predictive value; NPV, negative predictive value; *p*-value significant at <0.05.

## Data Availability

The datasets used and/or analyzed data during the current study are available from the corresponding author on reasonable request.

## Abbreviations

RSNA	Radiological Society of North America
RT-PCR	reverse transcription polymerase chain reaction
COVID-19	coronavirus disease 2019
PPV	positive predictive value
NPV	negative predictive value
SARS-CoV-2	severe acute respiratory syndrome coronavirus 2
GGO	ground-glass opacity
IRB	Institutional Review Board
STR	Society of Thoracic Radiology
ACR	American College of Radiology
TP	true positive
FP	false positive
TN	true negative
FN	false negative
CI	confidence interval

## References

[1] Naming the Coronavirus Disease (COVID-19) and the Virus That Causes It. https://www.who.int/emergencies/diseases/novel-coronavirus-2019/technical-guidance/naming-the-coronavirus-disease-(covid-2019)-and-the-virus-that-causes-it.

[2] (2020). Coronaviridae Study Group of the International Committee on Taxonomy of Viruses the Species Severe Acute Respiratory Syndrome-Related Coronavirus: Classifying 2019-NCoV and Naming It SARS-CoV-2. Nat. Microbiol..

[3] Zhu N., Zhang D., Wang W., Li X., Yang B., Song J., Zhao X., Huang B., Shi W., Lu R. (2020). A Novel Coronavirus from Patients with Pneumonia in China, 2019. N. Engl. J. Med..

[4] Wang D., Hu B., Hu C., Zhu F., Liu X., Zhang J., Wang B., Xiang H., Cheng Z., Xiong Y. (2020). Clinical Characteristics of 138 Hospitalized Patients With 2019 Novel Coronavirus–Infected Pneumonia in Wuhan, China. JAMA.

[5] WHO Chief Declares End to COVID-19 as a Global Health Emergency|UN News. https://news.un.org/en/story/2023/05/1136367.

[6] Lee D.W., Gardner R., Porter D.L., Louis C.U., Ahmed N., Jensen M., Grupp S.A., Mackall C.L. (2014). Current Concepts in the Diagnosis and Management of Cytokine Release Syndrome. Blood.

[7] Tan W., Zhao X., Ma X., Wang W., Niu P., Xu W., Gao G.F., Wu G. (2020). A Novel Coronavirus Genome Identified in a Cluster of Pneumonia Cases—Wuhan, China 2019–2020. China CDC Wkly..

[8] Chen N., Zhou M., Dong X., Qu J., Gong F., Han Y., Qiu Y., Wang J., Liu Y., Wei Y. (2020). Epidemiological and Clinical Characteristics of 99 Cases of 2019 Novel Coronavirus Pneumonia in Wuhan, China: A Descriptive Study. Lancet.

[9] Xu X.-W., Wu X.-X., Jiang X.-G., Xu K.-J., Ying L.-J., Ma C.-L., Li S.-B., Wang H.-Y., Zhang S., Gao H.-N. (2020). Clinical Findings in a Group of Patients Infected with the 2019 Novel Coronavirus (SARS-CoV-2) Outside of Wuhan, China: Retrospective Case Series. BMJ.

[10] Zhang N., Wang L., Deng X., Liang R., Su M., He C., Hu L., Su Y., Ren J., Yu F. (2020). Recent Advances in the Detection of Respiratory Virus Infection in Humans. J. Med. Virol..

[11] Lu R., Zhao X., Li J., Niu P., Yang B., Wu H., Wang W., Song H., Huang B., Zhu N. (2020). Genomic Characterisation and Epidemiology of 2019 Novel Coronavirus: Implications for Virus Origins and Receptor Binding. Lancet.

[12] Guan W., Ni Z., Hu Y., Liang W., Ou C., He J., Liu L., Shan H., Lei C., Hui D.S.C. (2020). Clinical Characteristics of Coronavirus Disease 2019 in China. N. Engl. J. Med..

[13] Yang Y., Yang M., Shen C., Wang F., Yuan J., Li J., Zhang M., Wang Z., Xing L., Wei J. (2020). Evaluating the Accuracy of Different Respiratory Specimens in the Laboratory Diagnosis and Monitoring the Viral Shedding of 2019-NCoV Infections 2020. medRxiv.

[14] Bai H.X., Hsieh B., Xiong Z., Halsey K., Choi J.W., Tran T.M.L., Pan I., Shi L.-B., Wang D.-C., Mei J. (2020). Performance of Radiologists in Differentiating COVID-19 from Viral Pneumonia on Chest CT. Radiology.

[15] Rubin G.D., Ryerson C.J., Haramati L.B., Sverzellati N., Kanne J.P., Raoof S., Schluger N.W., Volpi A., Yim J.-J., Martin I.B.K. (2020). The Role of Chest Imaging in Patient Management During the COVID-19 Pandemic: A Multinational Consensus Statement From the Fleischner Society. Chest.

[16] Wong H.Y.F., Lam H.Y.S., Fong A.H.-T., Leung S.T., Chin T.W.-Y., Lo C.S.Y., Lui M.M.-S., Lee J.C.Y., Chiu K.W.-H., Chung T.W.-H. (2020). Frequency and Distribution of Chest Radiographic Findings in Patients Positive for COVID-19. Radiology.

[17] Li Y., Xia L. (2020). Coronavirus Disease 2019 (COVID-19): Role of Chest CT in Diagnosis and Management. Am. J. Roentgenol..

[18] Bernheim A., Mei X., Huang M., Yang Y., Fayad Z.A., Zhang N., Diao K., Lin B., Zhu X., Li K. (2020). Chest CT Findings in Coronavirus Disease-19 (COVID-19): Relationship to Duration of Infection. Radiology.

[19] Ye Z., Zhang Y., Wang Y., Huang Z., Song B. (2020). Chest CT Manifestations of New Coronavirus Disease 2019 (COVID-19): A Pictorial Review. Eur. Radiol..

[20] Parry A.H., Wani A.H., Yaseen M., Dar M.I. (2020). Demystifying Pulmonary Vascular Complications in Severe Coronavirus Disease-19 Pneumonia (COVID-19) in the Light of Clinico-Radiologic-Pathologic Correlation. Thromb. Res..

[21] Pan Y., Guan H., Zhou S., Wang Y., Li Q., Zhu T., Hu Q., Xia L. (2020). Initial CT Findings and Temporal Changes in Patients with the Novel Coronavirus Pneumonia (2019-NCoV): A Study of 63 Patients in Wuhan, China. Eur. Radiol..

[22] Liu K.-C., Xu P., Lv W.-F., Qiu X.-H., Yao J.-L., Gu J.-F., Wei W. (2020). CT Manifestations of Coronavirus Disease-2019: A Retrospective Analysis of 73 Cases by Disease Severity. Eur. J. Radiol..

[23] Zhu Y., Gao Z.-H., Liu Y.-L., Xu D.-Y., Guan T.-M., Li Z.-P., Kuang J.-Y., Li X.-M., Yang Y.-Y., Feng S.-T. (2020). Clinical and CT Imaging Features of 2019 Novel Coronavirus Disease (COVID-19). J. Infect..

[24] Li X., Zeng W., Li X., Chen H., Shi L., Li X., Xiang H., Cao Y., Chen H., Liu C. (2020). CT Imaging Changes of Corona Virus Disease 2019(COVID-19): A Multi-Center Study in Southwest China. J. Transl. Med..

[25] Simpson S., Kay F.U., Abbara S., Bhalla S., Chung J.H., Chung M., Henry T.S., Kanne J.P., Kligerman S., Ko J.P. (2020). Radiological Society of North America Expert Consensus Statement on Reporting Chest CT Findings Related to COVID-19. Endorsed by the Society of Thoracic Radiology, the American College of Radiology, and RSNA–Secondary Publication. J. Thorac. Imaging.

[26] Das K.M., Lee E.Y., Enani M.A., AlJawder S.E., Singh R., Bashir S., Al-Nakshbandi N., AlDossari K., Larsson S.G. (2015). CT Correlation With Outcomes in 15 Patients With Acute Middle East Respiratory Syndrome Coronavirus. Am. J. Roentgenol..

[27] Silva C.F., Alegria J., Ramos C., Verdugo J., Diaz J.-C., Varela C., Barbe M. (2021). RSNA-STR-ACR Consensus Statement for COVID-19 CT Patterns: Interreader Agreement in 240 Consecutive Patients and Association With RT-PCR Status. J. Comput. Assist. Tomogr..

[28] Hu Q., Guan H., Sun Z., Huang L., Chen C., Ai T., Pan Y., Xia L. (2020). Early CT Features and Temporal Lung Changes in COVID-19 Pneumonia in Wuhan, China. Eur. J. Radiol..

[29] Zhou S., Wang Y., Zhu T., Xia L. (2020). CT Features of Coronavirus Disease 2019 (COVID-19) Pneumonia in 62 Patients in Wuhan, China. AJR Am. J. Roentgenol..

[30] Som A., Lang M., Yeung T., Carey D., Garrana S., Mendoza D.P., Flores E.J., Li M.D., Sharma A., McDermott S. (2020). Implementation of the Radiological Society of North America Expert Consensus Guidelines on Reporting Chest CT Findings Related to COVID-19: A Multireader Performance Study. Radiol. Cardiothorac. Imaging.

[31] Ciccarese F., Coppola F., Spinelli D., Galletta G.L., Lucidi V., Paccapelo A., De Benedittis C., Balacchi C., Golfieri R. (2020). Diagnostic Accuracy of North America Expert Consensus Statement on Reporting CT Findings in Patients Suspected of Having COVID-19 Infection: An Italian Single-Center Experience. Radiol. Cardiothorac. Imaging.

[32] Ai T., Yang Z., Hou H., Zhan C., Chen C., Lv W., Tao Q., Sun Z., Xia L. (2020). Correlation of Chest CT and RT-PCR Testing for Coronavirus Disease 2019 (COVID-19) in China: A Report of 1014 Cases. Radiology.

[33] Kim D., Quinn J., Pinsky B., Shah N.H., Brown I. (2020). Rates of Co-Infection Between SARS-CoV-2 and Other Respiratory Pathogens. JAMA.

[34] Rocha C.O., Prioste T.A.D., Faccin C.S., Folador L., Tonetto M.S., Knijnik P.G., Mainardi N.B., Borges R.B., Garcia T.S. (2021). Diagnostic Performance of the RSNA-Proposed Classification for COVID-19 Pneumonia versus Pre-Pandemic Controls. Braz. J. Infect. Dis..

[35] Dangis A., Gieraerts C., De Bruecker Y., Janssen L., Valgaeren H., Obbels D., Gillis M., Van Ranst M., Frans J., Demeyere A. (2020). Accuracy and Reproducibility of Low-Dose Submillisievert Chest CT for the Diagnosis of COVID-19. Radiol. Cardiothorac. Imaging.

[36] Jyoti D., Mehta V. (2022). Correlation between Chest CT and RT-PCR Testing in India’s Second COVID–19 Wave: A Retrospective Cohort Study. J. Assoc. Physicians India.

[37] Falaschi Z., Danna P.S.C., Arioli R., Pasché A., Zagaria D., Percivale I., Tricca S., Barini M., Aquilini F., Andreoni S. (2020). Chest CT Accuracy in Diagnosing COVID-19 during the Peak of the Italian Epidemic: A Retrospective Correlation with RT-PCR Testing and Analysis of Discordant Cases. Eur. J. Radiol..

[38] Fang Y., Zhang H., Xie J., Lin M., Ying L., Pang P., Ji W. (2020). Sensitivity of Chest CT for COVID-19: Comparison to RT-PCR. Radiology.

[39] Corman V.M., Landt O., Kaiser M., Molenkamp R., Meijer A., Chu D.K., Bleicker T., Brünink S., Schneider J., Schmidt M.L. (2020). Detection of 2019 Novel Coronavirus (2019-NCoV) by Real-Time RT-PCR. Euro Surveill..

[40] Kucirka L.M., Lauer S.A., Laeyendecker O., Boon D., Lessler J. (2020). Variation in False-Negative Rate of Reverse Transcriptase Polymerase Chain Reaction-Based SARS-CoV-2 Tests by Time Since Exposure. Ann. Intern. Med..

[41] Tahamtan A., Ardebili A. (2020). Real-Time RT-PCR in COVID-19 Detection: Issues Affecting the Results. Expert Rev. Mol. Diagn..

